# Correction: Recent developments of a co-immobilized laccase–mediator system: a review

**DOI:** 10.1039/d2ra90056d

**Published:** 2022-05-27

**Authors:** Yaohua Gu, Lin Yuan, Leina Jia, Ping Xue, Huiqin Yao

**Affiliations:** Key Laboratory of Environmental Factors and Chronic Disease Control, School of Public Health and Management, Ningxia Medical University Yinchuan 750004 China; College of Chemistry & Chemical Engineering, Ningxia University Yinchuan 750021 China ping@nxu.edu.cn; School of Basic Medical Sciences, Ningxia Medical University Yinchuan 750004 China huiqin_yao@163.com

## Abstract

Correction for ‘Recent developments of a co-immobilized laccase–mediator system: a review’ by Yaohua Gu *et al.*, *RSC Adv.*, 2021, **11**, 29498–29506, https://doi.org/10.1039/D1RA05104K.

The authors regret that an incorrect grant number was shown in the acknowledgements section of the published article. The corrected section should read:

This work was financially supported by the National Natural Science Foundation of China (No. 21663020) and Special talent start-up project of Ningxia Medical University (XT2020017).

The Royal Society of Chemistry apologises for these errors and any consequent inconvenience to authors and readers.

## Supplementary Material

